# Quality of life and pain in patients with thalidomide embryopathy in Japan

**DOI:** 10.1002/mgg3.1464

**Published:** 2020-09-06

**Authors:** Koubun Imai, Hanae Sone, Ken Otomo, Yuki Nakano, Fumihiko Hinoshita

**Affiliations:** ^1^ Department of Psychiatry Hitachi Medical Education and Research Center University of Tsukuba Hospital Ibaraki Japan; ^2^ Department of Psychiatry Center Hospital of the National Center for Global Health and Medicine Shinjyuku‐ku Japan; ^3^ Department of Nephrology Center Hospital of the National Center for Global Health and Medicine Shinjyuku‐ku Japan

**Keywords:** catastrophizing, mental health, pain, quality of life, thalidomide embryopathy

## Abstract

**Background:**

The aim of this study was to assess psychological/psychiatric problems and quality of life (QOL) in patients with thalidomide embryopathy (TE), with a specific focus on pain, including pain severity and the effects of coping strategies for pain.

**Methods:**

A questionnaire survey was conducted to evaluate the severity of pain experienced by patients with TE, pain management strategies, time perspective, mental health status, and QOL. Of 67 patients with TE who underwent a health checkup, 51 respondents who gave valid responses were included in analysis.

**Results:**

GHQ‐28 suggested that 41.2% of respondents appeared to potentially have psychiatric disorders. The mean scores of QOL were still within a normal range. There is no significant differences were found between limb disability group and hearing impairment group in QOL or mental health status. About 82.4% of respondents reported that they experience physical pain, and the use of the cognitive coping strategy “catastrophizing” to cope with pain was significantly associated with mental health status and QOL.

**Conclusion:**

This study demonstrate that although some patients with TE have some form of mental health problem, they still maintain a normal range QOL despite their disabilities. In addition, pain was not as strongly associated with mental health problems and QOL as would be expected, and variables such as “catastrophizing” to cope with pain appear to potentially be associated with reduced mental health and QOL.

## Graphical abstract

This study demonstrate that some patients with thalidomide embryopathy have some form of mental health problems, they still maintain a normal range QOL. Variables such as “catastrophizing” to cope with pain was associated with reduced mental health and QOL.

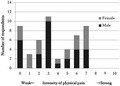

## 1 INTRODUCTION

Many patients with thalidomide embryopathy (TE) are now approaching middle age, and are anxious about issues such as their health and that of their family members, growing nursing care needs, and financial problems associated with retirement (Yoshizawa, Kimura, & Moriyoshi, 2012). Kruse et al. (2012) described how reduced physical function and pain experienced by TE Patients threatens their daily lives, explaining that the secondary sequelae (e.g., numbness and paralysis) and pain that develop with age, as well as associated difficulties with moving the body and physical fatigue, reduce the scope of activities they are capable of performing. A study conducted in England also noted that physical dimension of health related quality of life (QOL) in patients with TE is significantly lower than in the general population (Newbronner, Chamberlain, Borthwick, & Baxter, 2012). A Study conducted in Sweden report that physical dimension of health‐related QOL in TE patients was significantly lower than the general national population. However, no significant differences were found in mental aspect of QOL (Ghassemi Jahani, Karlsson, Brisby, & Danielsson, 2016). In Japan, Saito (2002) researched the mental health of TE patients and they reported that hearing impairment group has poor mental health comparing with limb deformity group. Also Imai et al. (2014) reported that 59% of TE patients who participated in the study were assessed as having some kind of mental health problems. However, there is no research on QOL.

As patients with TE continue to age, they will presumably experience further decline in physical function and increasing pain, with a corresponding drop in QOL. Therefore, this study was conducted to assess psychological/psychiatric problems and QOL in patients with TE, with a specific focus on the topic of pain, including pain severity and the effects of strategies for coping with pain.

## 2 METHODS

### 2.1 Participants

In this study, we recruited participants from TE patients who underwent a health check‐up carried out by TE research group which was organized by Japanese ministry of health, labour, and welfare. Check‐up was held in three different facilities, National Center for Global Health and Medicine, Teikyo University Hospital, and Kyoto Medical Center.

### 2.2 Procedure

A questionnaire was administered to patients with TE who are planning to have a health check‐up. Before their health check‐up, they were sent an explanation of the purpose of the study, a questionnaire form, and a consent form through the Ishizue Foundation (Thalidomide Welfare Center in Japan). No exclusion criteria were used. They brought the completed questionnaire form and consent form with them on the day of their health check‐up. Then, a study administrator gave another verbal explanation of the purpose and nature of the study, and collected written consent forms and questionnaire forms from only those candidates who consented to participate.

Before obtaining consent, care was taken to fully explain that there would be no negative consequences if they did not agree to participate in the survey. Of 67 patients with TE who underwent a health check‐up between 2014 and 2017, 51 participants have given consent that their data to be used in the research.

### 2.3 Survey contents

#### 2.3.1 Basic information sheet

Respondents were asked about their name, age, sex, diagnosed disability type, marital status, household composition, and employment status (in multiple choice format). They rated the severity of their pain using the Numerical Rating Scale (NRS). The first question was: “How would you rate your physical pain right now? Please circle the appropriate number.” Respondents were asked to circle a number on an 11‐point scale from “0. No pain at all” to “10. The worst pain imaginable.” (Haefeli & Elfering, 2006). Statistical analysis was performed using numerical ratings of pain. The next question, which was about the site of pain, was: “Where do you feel pain? Please write in the box below. You may write more than one part.” This was in a free‐response format.

#### 2.3.2 The General Health Questionnaire (GHQ‐28)

GHQ‐28 is a measure of mental health developed by Goldberg and Hillier (1979). It has been translated in 38 different languages and widely used in many studies (Jackson, 2007) with numerous populations including people with physical disease (Sterling, 2011). Because of these reasons, we used in our research. For another reason, In Japan, Studies about TE patients focused on Mental health was Conducted by Saito (2002) and those studies were used GHQ‐28. To take importance of follow‐up on a mental health into consideration, we chose same scale for our studies. Original GHQ‐28 was modified into a Japanese version by Nakagawa and Daibo (1985). It consists of the four essential scales for “somatic symptoms,” “anxiety and insomnia,” “social dysfunction,” and “depression” with a total of 28 items.

#### 2.3.3 The 36‐item Japanese version of the 36‐item Short Form Health Survey (SF‐36)

SF‐36 was used after applying for a use license. This Questionnaire was used to assess the QOL of participants. The SF‐36 consists of 35 items that assess eight health concepts (physical functioning, role limitations due to physical health, bodily pain, general health perceptions, energy/fatigue vitality, social functioning, role limitations due to emotional problems, and general health), and one individual item (health change) that assesses changes in health (Fukuhara & Suzukamo, 2004). The standard version measures QOL over the past month. These eight sub scales could aggregated into two measures: Physical component summary (PCS) and Mental component summary (MCS). Higher score represent better physical health and mental health. The “PCS” and “MCS” scores were used in analysis.

The SF‐36 has been standardized in men and women from their 20’s to 70’s in Japan (*N* = 2279). For the purposes of this study, PCS and MCS scores were calculated using the scoring algorithm developed by iHope International.

#### 2.3.4 Coping Strategy Questionnaire (CSQ)

The CSQ is a 16‐item scale that evaluates strategies for coping with pain. It is composed of two concepts: cognitive coping strategies (12 items) and behavioral coping strategies (4 items) (Otake & Shimai, 2002). The cognitive coping strategies consist of the six subcategories “praying/hoping” (2 items), “catastrophizing” (2 items), “self‐statements” (2 items), “diverting attention” (2 items), “reinterpretation of pain” (2 items), and “ignoring pain” (2 items), and the behavioral coping strategies consist of the two subcategories “increasing pain behaviors” (2 items) and “increasing activity levels” (2 items). Participants were asked, “How do you cope with the pain you are currently experiencing?” For each of the 16 coping strategies, they were asked to choose from 7 options ranging from “0. Never” to “6. Always.”

#### 2.3.5 Experiential Time Perspective Scale

Time perspective is defined as “the totality of the individual's views of his psychological future and psychological past existing as a given time” (Lewin, 1951). It is considered that time perspective influence on human behavior, attitude, and decision unrecognizably (Shimojima, Sato, & Ochi, 2012; Zimbardo & Boyd, 1999). Higata and Okamoto (2008) reported that there is an association between Time perspective and mental health in middle‐age people in Japan. Therefore, we conducted an investigation for this association in TE patients. In this study, we used Experiential Time Perspective Scale developed by Shirai (1994) to assess the way of thinking or feeling of participants, about their past, present, and future. This 18‐item scale consists of the 4 factors “hopefulness” (5 items), “goal‐directedness” (5 items), “self‐fullness” (4 items), and “acceptance of the past” (4 items). For each item, respondents were asked to choose from five options ranging from “0. Not at all true” to “6. True.”

### 2.4 Statistical analysis

All statistical analyses were performed using IBM SPSS Statistics version 25.

## 3 RESULTS

### 3.1 Descriptive statistics

Of the 67 patients with TE who had a health check‐up during the study period, 51 submitted a valid response. Of these 51 participants (30 men and 21 women, mean age 53.6 years, SD = 1.50), 23 were from the Center Hospital of the National Center for Global Health and Medicine (18 men and 5 women), 9 were from Teikyo University Hospital (4 men and 5 women), and 19 were from Kyoto Medical Center (8 men, 11 women).

Reported disabilities were limb disability in 37 respondents (72.55%, 20 men and 17 women) and hearing impairment in 14 respondents (27.45%, 10 men and 4 women). All respondents with limb disability had upper body disability, and 2 (1 man and 1 woman) had both upper and lower body disability. No respondent had concurrent limb disability and hearing impairment.

Twenty‐seven respondents (52.9%) were married. When asked about household composition and employment status, 37 respondents (72.5%) reported living with their family. Thirty‐six (70.6%) were engaged in some form of employment, 4 (7.8%) were unemployed or on leave, 7 (13.7%) were homemakers, and 4 (7.8%) answered “Other.”

### 3.2 Pain severity

**FIGURE 1:**
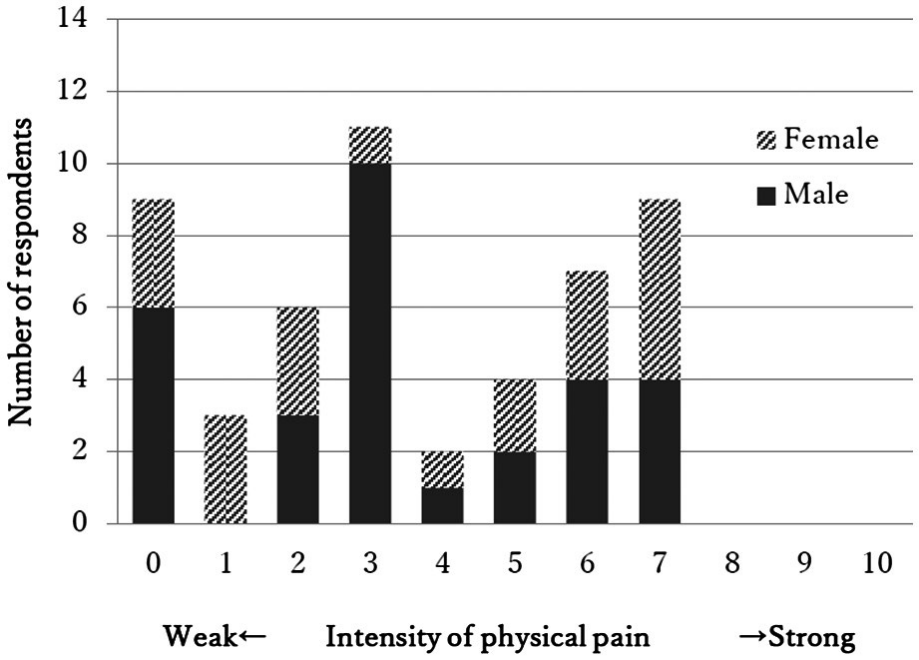
NRS was used to assess pain. In this scale, participants are asked to circle the number between 0 and 10 which best describes their subjective pain. Nine participants (17.6%) reported no pain. Other 42 participants (82.4%) rated their subjective pain in 1 to 10, and 7 was the highest pain

Pain severity reported by participants (*n* = 51) were demonstrated in Figure 1. Nine TE patients (17.6%) reported no physical pain and 42 (82.4%) reported physical pain. The scale “7” was the highest pain reported in this study. Most frequently reported pain severity was “3” by men and “7” by women.

### 3.3 Sites of pain

The most common site of pain reported (in free‐response format) was the shoulders (*n* = 24, 47.1%), followed by the lower back (*n* = 21, 41.2%) and the neck (*n* = 16, 31.4%) (Table 1).

**TABLE 1:** Sites of pain (*N* = 51, multiple response)

Body part	Number of respondents
Shoulders	24 (47.1%)
Lower back	21 (41.2%)
Neck	16 (31.4%)
Fingers	12 (23.5%)
Arms	11 (21.6%)
Back	11 (21.6%)
Knees	7 (13.7%)
Hips	5 (9.8%)
Femur	2 (3.9%)
Eyes, dentures, elbow, stomach, intestines, ankle, head, chest, heart	1 each (2.0%)

### 3.4 GHQ‐28, SF‐36, CSQ, Experimental Time Perspective

Means and standard deviations of scales were calculated (Table 2). The mean total GHQ score was 6.27 (SD = 5.61).The mean GHQ score was over the cut off of 6, and individual results suggested that 21 respondents (41.2%) potentially had psychiatric disorders. By disability type, the number of respondents with a score over the cutoff was 15 (40.5%) for the limb disability group and 8 (57.1%) for the hearing impairment group.

**TABLE 2:** Means and standard deviations (SD) of scales

	*n* = 51	
	Mean	SD
GHQ‐28		
Total score	6.27	5.61
Somatic symptoms	2.12	2.06
Anxiety/insomnia	2.20	2.09
Social dysfunction	1.02	1.58
Depression	0.94	1.86
SF‐36		
Physical functioning	48.99	9.83
Role limitations due to physical health	47.97	9.34
Physical pain	45.23	9.56
General health perceptions	42.38	7.67
Energy/fatigue	47.00	10.27
Social functioning	49.67	10.10
Role limitations due to emotional problems	48.15	9.05
General mental health	48.51	9.91
Physical component summary	48.27	9.67
Mental component summary	46.41	9.26
CSQ		
Cognitive coping strategies		
Praying/hoping	3.88	3.83
Catastrophizing	1.84	2.63
Self‐statements	4.29	4.10
Diverting attention	3.65	3.80
Reinterpretation of pain	2.27	2.94
Ignoring pain	2.75	2.99
Behavioral coping strategies		
Increasing pain behavior	5.59	3.85
Increasing activity levels	5.88	3.79
Experiential Time Perspective Scale		
Self‐fullness	17.41	3.56
Goal‐directedness	15.73	4.55
Acceptance of the past	14.27	3.56
Hopefulness	13.59	3.62

Abbreviations: CSQ, Coping Strategy Questionnaire; GHQ‐28, The General Health Questionnaire; SF‐36, The 36‐item Japanese version of Short Form Health Survey.

The PCS and MCS are normalized to a mean of 50 (SD = 9.8), mean scores for study participants were 48.27 (SD = 9.67) for PCS and 46.41 (SD = 9.26) for MCS.

### 3.5 Comparison by disability type

Student's *t* test was used to compare pain severity, GHQ, SF‐36, CSQ, and Experiential Time Perspective Scale between hearing impairment group and limb disability group. The hearing impairment group had significantly higher scores than the limb disability group for “diverting attention (*t* (49) = −2.13, *p* < 0.05)” and “reinterpretation of pain (*t* (49) = −2.74, *p* < 0.05)” on the CSQ.

However, there were no significant differences in any other parameter such as pain severity, mental health, or QOL between disability types (Table 3).

**TABLE 3:** Comparison of score of scales between limb disability group and hearing impairment group by using *t* test

	Limbs (*N* = 37)	Hearing (*N* = 14)	df	*p* value
	Mean (SD)	Mean (SD)		
Pain severity	3.41 (2.39)	3.93 (2.79)	49	0.51
GHQ				
Total score	5.86 (5.62)	7.36 (5.64)	49	0.40
Somatic symptoms	2.05 (1.93)	2.29 (2.43)	49	0.72
Anxiety/insomnia	1.86 (2.04)	3.07 (2.02)	49	0.06
Social dysfunction	1.11 (1.71)	0.79 (1.19)	49	0.52
Depression	0.84 (1.86)	1.21 (1.89)	49	0.52
SF‐36				
Physical component summary	47.60 (1.00)	50.04 (8.82)	49	0.43
Mental component summary	46.91 (8.49)	45.10 (11.29)	49	0.54
CSQ				
Cognitive coping strategies				
Praying/hoping	3.38 (3.68)	5.21 (4.04)	49	0.13
Catastrophizing	1.59 (2.44)	2.50 (3.08)	49	0.28
Self‐statements	3.62 (3.79)	6.07 (4.48)	49	0.06
Diverting attention	2.97 (3.40)	5.43 (4.36)	49	**0.04** *
Reinterpretation of pain	1.62 (2.27)	4.00 (3.82)	49	**0.04** *
Ignoring pain	2.24 (2.37)	4.07 (4.03)	49	0.13
Behavioral coping strategies				
Increasing pain behavior	6.03 (3.97)	5.50 (3.39)	49	0.66
Increasing activity levels	5.70 (4.12)	5.29 (3.17)	49	0.73
Experiential Time Perspective Scale				
Self‐fullness	17.08 (3.77)	18.29 (2.87)	49	0.29
Goal‐directedness	15.22 (4.67)	17.07 (4.07)	49	0.20
Acceptance of the past	14.46 (3.73)	13.79 (3.12)	49	0.55
Hopefulness	13.08 (3.83)	14.93 (2.70)	49	0.10

Abbreviations: CSQ, Coping Strategy Questionnaire; GHQ‐28, The General Health Questionnaire; SF‐36, The 36‐item Japanese version of the Short Form Health Survey.

Significant results are indicated in bold (*p* < 0.05).

* *p* < 0.05,

** *p* < 0.01.

### 3.6 Correlations between variables

Correlations between pain severity, GHQ‐28, SF‐36, CSQ, and the Experiential Time Perspective Scale were analyzed using Pearson's Correlation Analysis (Table 4).

**TABLE 4:** Correlations of pain severity, GHQ‐28, SF‐36, CSQ, and Experiential Time Perspective Scale

	Pearson's correlation coefficient		PCS	(*N* = 51)
	Pain severity	Total GHQ‐28 score		MCS
Pain severity	**—**			
GHQ‐28				
Total score	0.18	**—**		
SF‐36				
Physical component summary	**−0.32** *	−0.13	**—**	
Mental component summary	−0.20	**−0.69** **	0.04	**—**
CSQ				
Cognitive coping strategies				
Praying/hoping	**0.51** **	0.26	−0.26	−0.08
Catastrophizing	**0.51** **	**0.50** **	**−0.33** *	**−0.41** **
Self‐statements	0.26	0.23	−0.26	−0.05
Diverting attention	0.24	0.08	−0.12	0.02
Reinterpretation of pain	**0.28** *	0.16	−0.04	−0.16
Ignoring pain	**0.30** *	**0.31** *	−0.07	−0.20
Behavioral coping strategies				
Increasing other behavior	0.17	0.13	0.19	−0.21
Increasing activity levels	0.26	0.05	0.21	−0.21
Experiential Time Perspective Scale				
Self‐fullness	0.06	**−0.50** **	**0.29** *	**0.38** **
Goal‐directedness	−0.16	**−0.35** *	**0.39** **	**0.29** *
Acceptance of the past	0.05	−0.27	−0.10	0.24
Hopefulness	−0.15	**−0.40** **	**0.35** *	**0.37** **

Abbreviations: CSQ, Coping Strategy Questionnaire; GHQ‐28, The General Health Questionnaire; SF‐36, The 36‐item Japanese version of the Short Form Health Survey

Significant results are indicated in bold (*p* < 0.05).

* *p* < 0.05,

** *p* < 0.01.

Significant correlations with pain severity were observed for “PCS” on the SF‐36 (*r* = −0.32, *p* < 0.05) and for “praying/hoping” (*r* = 0.51, *p* < 0.01), “catastrophizing” (*r* = 0.51, *p* < 0.01), “reinterpretation of pain” (*r* = 0.28, *p* < 0.05), and “ignoring pain” (*r* = 0.30, *p* < 0.05) on the CSQ. There were no significant correlations with total GHQ score, MCS, or the Experiential Time Perspective Scale.

Total GHQ score was correlated with “MCS” on the SF‐36 (*r* = −0.69, *p* < 0.01), “catastrophizing” (*r* = 0.50, *p* < 0.01) and “ignoring pain” (*r* = 0.31, *p* < 0.05) on the CSQ, and “present satisfaction” (*r* = −0.5, *p* < 0.01), “goal‐directedness” (*r* = −0.35, *p* < 0.05), and “hope” (*r* = −0.4, *p* < 0.01) on the Experiential Time Perspective Scale.

“Catastrophizing” on the CSQ showed a positive correlation with pain severity and total GHQ score, and a negative correlation with “PCS” and “MCS” on the SF‐36. The only correlation between the CSQ and the QOL measure SF‐36 was for “catastrophizing.”

The “self‐fullness,” “goal‐directedness,” and “hopefulness” items of the Experiential Time Perspective Scale showed a negative correlation with total GHQ score and a positive correlation with “PCS” and “MCS” on the SF‐36. “Acceptance of the past” was not correlated with any scale.

### 3.7 Multiple regression analysis

To determine which factors affect total GHQ score and “PCS” and “MCS” on the SF‐36, stepwise multiple regression analysis was performed with each of these three items set as the dependent variable and various parameters such as age, sex, disability type, CSQ, and Experiential Time Perspective Scale results set as independent variables (Table 5).

**TABLE 5:** Results of stepwise regression analysis that set GHQ, PCS and MCS as dependent variables

Dependent variable	Independent variable	SE	*β*	*p* value	VIF	*R* ^2^	*F*
GHQ‐28	Catastrophizing	**0.24**	**0.41**	**0.00** **	1.05	**0.38**	**16.43** **
	Self‐fullness	**0.18**	**−0.41**	**0.00** **	1.05		
Physical component summary of SF‐36	Catastrophizing	**0.47**	**−0.36**	**0.00** **	1.11	**0.25**	**6.40** **
	Goal‐directedness	**0.27**	**0.30**	**0.02** *	1.05		
	Increasing pain behaviour	**0.33**	**0.26**	**0.049** *	1.11		
Mental component summary of SF‐36	Catastrophizing	**0.45**	**−0.35**	**0.00** **	1.05	**0.22**	**8.07** **
	Self‐fullness	**0.33**	**0.29**	**0.02** *	1.05		

Abbreviations: GHQ‐28, The General Health Questionnaire; SF‐36, The 36‐item Japanese version of the Short Form Health Survey.

Significant results are indicated in bold (p < 0.05).

* *p* < 0.05,

** *p* < 0.01.

When total GHQ score was set as the dependent variable, it was associated with “catastrophizing” (*β* = 0.41, *p* < 0.01) and “self‐fullness” (*β* = −0.41, *p* < 0.01) (*R^2^* = 0.57, *p* < 0.01).

When “PCS” on the SF‐36 was set as the dependent variable, it was associated with “catastrophizing” (*β* = −0.36, *p* < 0.01), “goal‐directedness” (*β* = 0.30, *p* < 0.05), and “increasing pain behavior” (*β* = 0.26, *p* < 0.05) (*R* ^2^ = 0.25, *p* < 0.01).

When “MCS” on the SF‐36 was set as the dependent variable, it was associated with “catastrophizing” (*β* = −0.35, *p* < 0.01) and “self‐fullness” (*β* = 0.29, *p* < 0.05) (*R^2^* = 0.22, *p* < 0.01).

## 4 DISCUSSION

Results of the analysis in this study suggested that 41.2% of participating TE patients may have some form of mental health problem. According to the study conducted in Germany, the Structured Clinical Interview for DSM‐IV (SCID) showed that the four‐week prevalence of mental disorders in TE patients were 47.2% and it was almost twice as high as the general population (Niecke et al., 2017). These finding shows that there is a high risk of mental health problem in TE patients. However, SF‐36 results showed that general QOL was within the normal range, indicating that these patients with TE are still maintaining normal range QOL despite their disabilities. Similar results were reported by study conducted in Sweden. Ghassemi Jahani et al. (2016) reported that the physical aspects of QOL in many of individuals were significantly lower than those of the national reference population, but that the mental aspects of QOL were not significantly affected.

Saito (2002) compared patients with TE with different types of disability and they found that total GHQ score was higher in the hearing impaired group in 2000. In this study, however, there was no significant difference between groups. GHQ‐28 is one of optimal measures for case identification but not sufficiently accurate as a definitive case‐finding tool (Meader et al., 2011). Several study showed that standardized questionnaire has limited impact or usefulness on screening or detection (Christensen et al., 2003; Gilbody, Sheldon, & House, 2008). In addition, this questionnaire has been available for many years, and therefore old now. For these reasons, our study findings brought by using screening questionnaire must be interpreted with caution. In the future study, using structured interview (e.g., M.I.N.I., SCID) may solve this problem.

In the relation of pain and QOL, Ghassemi Jahani et al. (2016) reported that there was a correlation between physical QOL and measured pain. Moreover, they reported that mental QOL was not correlated with pain subscale. In our study, similar result was found that pain severity and Physical QOL were significantly correlated but no significant correlation was found between pain severity and mental QOL. Furthermore, in our study, we conducted a multiple regression analysis. For the result, the cognitive strategy “catastrophic thinking” for coping with pain was significantly associated with total GHQ score, Physical QOL and Mental QOL. However, pain severity had no significant regression weight. This tendency toward “catastrophizing” has been shown to intensify pain and increase mental distress (Sullivan et al., 2001). The “self‐fullness” subcategory of the Experiential Time Perspective Scale, which includes items such as “I am fulfilled in my daily life” and “I am satisfied with my current life,” was significantly associated with total GHQ score and mental QOL. This suggests that fulfillment and satisfaction in daily life may be critically significant to the mental health of middle‐aged TE patients.

Although the patients with TE who participated in this study had QOL within a normal range, studies conducted in Germany, England, and Sweden have found that physical QOL in patients with TE is significantly lower than in the general population (Ghassemi Jahani et al., 2016; Kruse et al., 2012; Newbronner et al., 2012). One reason for the difference in results may be that this study only included people who were able to visit a hospital for a health checkup, and these subjects may not be representative of the entire population of patients with TE in Japan. Inclusion of responses from patients with TE unable to visit a hospital would have ensured that the survey results better reflected the real conditions of this group. In order to accomplish this, it will be necessary to consider questionnaire distribution methods, questionnaire content, and analytical methods in order to provide support to patients with TE that takes into account physical changes, psychological changes, and financial problems associated with aging as well as aspects of their social environment such as range of movement and mobility.

## 5 CONCLUSION

This study demonstrate that although some patients with TE have some form of mental health problems, they still maintain a normal range QOL despite their disabilities. In addition, pain was not as strongly associated with mental health problems and QOL as would be expected, and variables such as “catastrophizing” to cope with pain appear to potentially be associated with reduced mental health and QOL.

## CONFLICT OF INTEREST

The authors declare no conflict of interest.

## AUTHOR’S CONTRIBUTIONS

K.I. and H.S. designed the study. H.S., K.O., and Y.N. contributed to the investigation and data curation. K.O. conducted statistical analyses. All authors discussed the results and K.O. drafted and edited the manuscript. K.I. provided critical feedback and shaped the manuscript. F.H. acquired the financial support for the project and supervised the project. All authors reviewed the final manuscript.
